# Apolipoprotein B/AI ratio as an independent risk factor for intracranial atherosclerotic stenosis

**DOI:** 10.18632/aging.102216

**Published:** 2019-09-03

**Authors:** Yan Sun, Xiao-He Hou, Dong-Dong Wang, Ya-Hui Ma, Chen-Chen Tan, Fu-Rong Sun, Mei Cui, Qiang Dong, Lan Tan, Jin-Tai Yu

**Affiliations:** 1 Department of Neurology, Qingdao Municipal Hospital, Qingdao University, Qingdao, China; 2 Department of Neurology and Institute of Neurology, Huashan Hospital, Shanghai Medical College, Fudan University, Shanghai, China

**Keywords:** apolipoprotein AI, apolipoprotein B, intracranial atherosclerotic stenosis, ischemic stroke, steno-occlusion lesions

## Abstract

To investigate the relation of higher apolipoprotein B/apolipoprotein AI (apoB/AI) ratio with the risk of suffering intracranial atherosclerotic stenosis (ICAS) in both stroke and non-stroke population, we enrolled 1138 patients with acute ischemic stroke (359 with ICAS, 779 without ICAS) and 1072 non-stroke controls (239 with ICAS, 833 without ICAS) into the study. ICAS was defined as atherosclerotic stenosis >50% or the occlusion of the several main intracranial arteries. ApoB/AI ratio of patients with ICAS was significantly higher than those of individuals without ICAS in both stroke group and non-stroke groups. Increased ratio of apoB/AI was an independent risk factor for ICAS in both stroke group (OR 2.80, 95% CI 1.45-5.42, *p*=0.002) and non-stroke groups (OR 3.38, 95% CI 1.61-7.12, *p*<0.001). Compared with the lowest quartile, the third (Stroke OR=1.71, 95%CI, 1.11-2.63, *p=*0.014; Non-stroke OR=1.71, 95%CI, 1.04-2.82, *p*=0.033) and forth quartiles (Stroke OR=2.06, 95%CI, 1.27-3.35, *p*=0.003; Non-stroke OR=2.00, 95%CI, 1.16-3.49, *p*=0.012) were independent risk factors for ICAS in both stroke (*p* value for trend=0.001)) and non-stroke (*p* value for trend=0.006) groups. In summary, increased apoB/AI ratio was a valuable independent risk factor for ICAS in stroke patients as well as in non-stroke controls.

## INTRODUCTION

Stroke event is the second leading cause of death worldwide and the leading cause of both chronic disability and long-term institutionalization [1]. Ischemic stroke accounts for more than 80% of all acute stroke events [2, 3]. It’s widely accepted that intracranial atherosclerotic stenosis is one of the major risk factors for ischemic stroke and about 80–97% of the population over age 65 years have pathological evidence of intracranial atherosclerosis [4]. ICAS is the most common cause of stroke in Asian population [4]. According to the CICAS study (The Chinese Intracranial Atherosclerosis Study), a large, multicenter, prospective study in China, has mentioned that the prevalence of intracranial atherosclerotic stenosis accounts for 33% to 67% of stroke or transient ischemic attack (TIA) cases in China and other countries in Asia [5]. The high incidence of stroke imposes a heavy economic burden on society, preventing ICAS and identifying the risk factors of ICAS become crucial.

ICAS was highly associated with advanced age, metabolic syndrome, diabetes mellitus, hypertension and dyslipidemia [6]. But investigations about whether apoB/AI ratio is an independent risk factor for ICAS still rarely. The apoB/AI ratio is a well-established indicator which reflects the balance between atherogenic apoB lipoprotein particles (mainly transform low-density lipoprotein cholesterol, LDL-c) and antiatherogenic apoAI lipoprotein particles (mainly transform high-density lipoprotein cholesterol, HDL-c) [7, 8]. Disruption of the balance may provoke the progression of atherosclerosis and cause ischemic stroke. Previous studies have also reported that acute ischemic stroke is associated with increased apoB/AI ratio in plasma [2, 9–13] and a high ratio of apoB/AI can also accelerate the increase in artery intima-media thickness and the risk of atherosclerosis [10, 14].

However, studies on apoB/AI ratio and ICAS in Asian population are still rare [15]. A previous South Korean study [16] has demonstrated that an increased apoB/AI ratio was an independent predictor for intracranial atherosclerotic stenosis in ischemic stroke patients. But whether this association still exists among non-stroke individuals hasn’t been proven. It is more essential to identify the modifiable risk factors of ICAS for non-stroke individuals so that preventive measures could be taken before the occurrence of stroke. We carried out the present study to examine whether this similar relationship between apoB/AI ratio and ICAS could be established amongst the stroke and non-stroke individuals in Chinese population.

## RESULTS

### Participant characteristics

**Figure 1:**
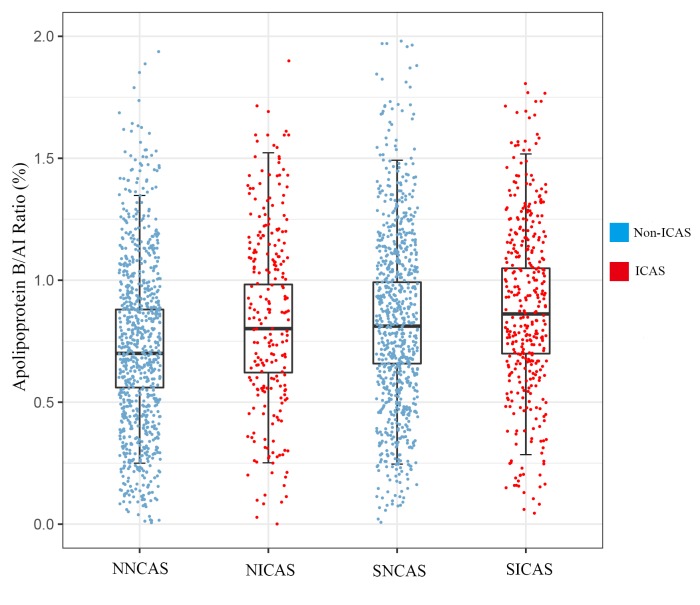
**The level of apoB/AI ratio and distribution of participants.** Data are represented as mean±SD; ICAS, intracranial atherosclerotic stenosis. SICAS, stroke with ICAS; SNCAS, stroke without ICAS; NICAS, non-stroke with ICAS; NNCAS, non-stroke without ICAS.

A total of 2210 participants were included in the study, among whom 1138 patients with acute ischemic stroke (359 with ICAS, 779 without ICAS) and 1072 non-stroke controls (239 with ICAS, 833 without ICAS). The apoB/AI ratio was significantly increased among patients with ICAS (mean 0.86±0.27, range 0.25-1.74) compared with patients without ICAS (mean 0.78±0.26, range 0.02-1.78, p<0.001). Baseline characteristics of all the participants are summarized in Table 1. As the results showed, in both stroke and non-stroke groups, age, sex, weight and BMI were not significant when compared patient with ICAS and without ICAS. There is no difference in apoAI level, but apoB level and the apoB/AI ratio differed across the SICAS and SNCAS groups. There are statistic significances in the presence of hypertension and level of glucose between the SICAS and SNCAS groups. In the comparison of the NICAS and NNCAS groups, the apoAI level, apoB level and their ratio showed apparent differences. Other significant factors including presence of hypertension, diabetes, level of glucose, HDL-c and smoking. The tests of interactions between HDL-c, LDL-c and apoB/AI ratio appeared non-significant results (In stroke group, HDL-c *p* for interaction=0.246, LDL-c *p* for interaction=0.517; In non-stroke group, HDL-c *p* for interaction=0.774, LDL-c *p* for interaction=0.211). The SICAS (0.88±0.26) group had the highest apoB/AI ratio, followed by the SNCAS (0.84±0.26) group, the NICAS (0.82±0.27) group, and lastly the NNCAS (0.73±0.24) group. The discrepancies and the participant distribution among the 4 groups were recorded in Figure 1.

**Table 1:** Baseline characteristics and ApoB/AI ratios among groups.

	**All participants With stroke (n=1138)**	**Stroke group**			**All participants Without stroke (n=1072)**	**Non-stroke group**			P*
		**SICAS (n=359)**	**SNCAS (n=779)**	***p***		**NICAS (n=239)**	**NNCAS (n=833)**	***P***	
Age	68.09±12.01	68.74±12.49	67.79±11.78	0.1272	69.9±10.19	70.68±11.08	69.73±9.92	0.1641	<0.001
Female	405(35.58%)	138(38.44%)	267(34.27%)	0.1726	510(47.57%)	113(47.28%)	397(47.65%)	0.9177	<0.001
Weight (Kg)	67.98±10.98	68.02±10.88	67.95±11.03	0.8617	65.60±9.48	66.02±10.28	65.47±9.24	0.9611	<0.001
BMI-Kg/m^2^	24.00±3.61	24.05±3.46	23.98±3.68	0.9462	23.63±2.96	23.74±3.38	23.60±2.83	0.5647	0.0796
Hypertension	852(74.86%)	288(80.22%)	564(72.40%)	0.0046	781(72.85%)	199(83.26%)	582(69.86%)	<0.001	0.2814
Smoke	436(38.31%)	127(35.37%)	309(39.66%)	0.1665	266(24.81%)	73(30.16%)	193(23.34%)	0.0199	<0.001
Drinking	327(28.73%)	101(28.13%)	226(29.01%)	0.7610	183(17.07%)	50(20.66%)	133(15.93%)	0.0727	<0.001
Diabetes	397(34.88%)	138(38.44%)	259(33.24%)	0.0876	316(29.47%)	90(38.01%)	226(27.21%)	0.0016	0.0065
Glucose,mg/dl	6.48±2.77	6.80±2.87	6.33±2.27	0.0010	5.79±2.02	6.19±2.31	5.68±1.91	0.0027	<0.001
HDL-c, mg/dl	1.14±0.34	1.12±0.29	1.16±0.39	0.0883	1.19±0.38	1.11±0.29	1.21±0.39	<0.001	<0.001
LDL-c, mg/dl	3.15±0.92	3.14±0.94	3.15±0.91	0.9475	2.96±0.90	3.01±0.96	2.94±0.88	0.3836	<0.001
ApoB, mg/dl	1.03±0.28	1.05±0.27	1.03±0.29	0.0425	0.95±0.26	0.99±0.27	0.94±0.25	0.0089	<0.001
ApoA-I, mg/dl	1.25±0.28	1.24±0.28	1.26±0.28	0.2613	1.31±0.27	1.25±0.26	1.32±0.26	<0.001	<0.001
ApoB/ApoA-I	0.85±0.26	0.88±0.26	0.84±0.26	0.0040	0.75±0.25	0.82±0.27	0.73±0.24	<0.001	<0.001

Total 1138 patients with ischemic stroke, 359 patients with intracranial atherosclerotic stenosis (ICAS), 779 without ICAS.

Total 1072 subjects in non-stroke group, 239 subjects with ICAS while 833 without ICAS.

Values provided are number (%) or mean±SD (standard deviation). *p* values are from one-way analysis of variance.

Apo, apolipoprotein; SICAS, stroke with ICAS; SNCAS, stroke without ICAS; NICAS, non-stroke with ICAS; NNCAS, non-stroke without ICAS; LDL-c, low-density lipoprotein cholesterol; HDL-c, high-density lipoprotein cholesterol; BMI, body mass index;

p* for comparisons between stroke and non-stroke groups

### Association of apoB/AI ratio and ICAS in stroke group

All covariates were incorporated into the multivariable logistic regression analysis of the SICAS and SNCAS groups. Findings of multivariable analysis to examine the association between apoB/AI ratio and ICAS are provided in Table 2. After adjusting these covariates, the apoB/AI ratio (OR 2.80, 95%CI 1.45-5.42, *p* =0.002) was demonstrated to be an independent risk factor for ICAS against a background of stroke. Other risk factors included the presence of hypertension (OR 1.52, 95%CI 1.12-2.08, *p* =0.008) and high level of glucose (OR 1.05, 95%CI 1.01-1.10, *p* =0.015).

**Table 2:** Crude, adjusted ORs and 95% confidence intervals for predictors of ischemic stroke and intracranial artery stenosis in groups using univariate and multiple logistic regression analyses.

	**Total^a^ (n=2210)**			**Stroke group^b^** **(n=1138)**			**Non-stroke group^c^ (n=1072)**		
	**Univariate OR**	**Multi-OR 95%CI**	***p***	**Univariate OR**	**Multi-OR 95%CI**	***p***	**Univariate OR**	**Multi-OR 95%CI**	***p***
Age	0.98	0.99(0.98-1.00)	0.1341	1.00	1.00(0.99-1.02)	0.2718	1.00	1.01(0.99-1.03)	0.0698
Female	0.60	0.73(0.57-0.93)	0.0112	1.19	1.30(0.88-1.96)	0.1985	0.99	1.48(0.91-2.44)	0.1150
Weight (Kg)	1.02	0.99(0.98-1.01)	0.7549	1.01	1.01(0.99-1.03)	0.4781	1.00	1.00(0.97-1.04)	0.6160
BMI-Kg/m^2^	1.01	1.05(1.00-1.09)	0.0234	1.00	0.97(0.90-1.04)	0.5770	1.01	0.99(0.89-1.10)	0.9237
Hypertension	1.10	1.12(0.91-1.36)	0.2612	1.54	1.52(1.12-2.08)	0.0075	2.14	1.91(1.31-2.82)	<0.001
Smoke	1.88	1.25(0.97-1.62)	0.0749	0.83	0.86(0.60-1.22)	0.4033	1.45	1.52(0.94-2.45)	0.0825
Dringking	1.95	1.38(1.06-1.81)	0.0160	0.95	1.20(0.84-1.71)	0.3135	1.39	1.25(0.75-2.07)	0.3779
Diabetes	1.28	1,23(1.02-1.48)	0.0283	1.25	1.21(0.92-1.57)	0.1579	1.62	1.31(0.95-1.79)	0.0961
Glucose, mg/dl	1.12	1.10(1.06-1.15)	<0.001	1.06	1.05(1.01-1.10)	0.0153	1.11	1.08(1.01-1.16)	0.0205
HDL-c, mg/dl	0.70	0.97(0.73-1.28)	0.8496	0.68	0.92(0.59-1.44)	0.7340	0.38	0.59(0.32-1.00)	0.0740
LDL-c, mg/dl	1.25	1.07(0.95-1.22)	0.2326	0.98	0.83(0.68-1.02)	0.0823	1.07	0.94(0.75-1.15)	0.5670
ApoB/AI ratio quartiles	4.25	3.35(2.16-5.23)	<0.001	1.80	2.80(1.45-5.42)	0.0020	3.91	3.38(1.61-7.12)	<0.001
1^st^	reference			reference			reference		
2^nd^	1.36	1.32(1.02-1.69)	0.0304	1.09	1.22(0.81-1.82)	0.3107	1.11	1.15(0.71-1.86)	0.5483
3^rd^	2.04	1.90(1.43-2.48)	<0.001	1.44	1.71(1.11-2.63)	0.0141	1.76	1.71(1.04-2.82)	0.0331
4^th^	2.88	2.53(1.82-3.44)	<0.001	1.59	2.06(1.27-3.35)	0.0034	2.30	2.00(1.16-3.49)	0.0125
*p* value for trend						0.0018			0.0060

SICAS, stroke with ICAS; SNCAS, stroke without ICAS; NICAS, non-stroke with ICAS; NNCAS, non-stroke without ICAS; LDL-c, low-density lipoprotein cholesterol; HDL-c, high-density lipoprotein cholesterol; BMI, body mass index; OR, odds ratio; CI, confidence interval; Multi-OR, multivariate OR;

Total^a^, between stroke and non-stroke groups; Stroke group^b^, between SICAS and SNCAS groups; Non-stroke group^c^, between NICAS and NNCAS groups

Participants were stratified into quartiles according to the distribution of their serum apoB/AI ratio. (Supplementary Figure 2A showed the distribution of subjects according to quartiles in four groups). For the SICAS and SNCAS groups, the regression analyses indicated that patients with higher apoB/AI ratio quartiles were more inclined to suffer ICAS, compared with the first quartile. Specifically, with the first quartile as the reference category, the ORs and 95%CI were 1.22(0.81-1.82) in the second quartile, 1.71(1.11-2.63) in the third quartile and 2.06(1.27-3.35) in the fourth quartile. The third (*p*=0.014) and forth (*p* =0.003) apoB/AI ratio quartiles were significantly associated with SICAS. We also conducted the trend test and final got the *p* value for trend (*p* =0.002) which was significant and it also indicated a dose-response relationship between apoB/AI ratio and the risk of suffering ICAS.

### Association of apoB/AI ratio and ICAS in non-stroke group

In NICAS and NNCAS groups, the regression analysis showed that the apoB/AI ratio (*p* <0.001) was still a significant risk factor of suffering ICAS. When referenced to the first quartile, ORs and 95%CI in the second quartile were 1.15(0.71-1.86), in the third quartile were 1.71(1.04-2.82) and in the fourth quartile were 2.00(1.16-3.49). The third (*p* =0.033) and fourth (*p* =0.012) quartiles were different between the two groups, which supports the apoB/AI ratio was an independent risk factor for ICAS. The *p* value for trend (*p*=0.006) was still remarkable in non-stroke population. Similar to stroke groups, the presence of hypertension (OR 1.91, 95%CI 1.31-2.82, *p* <0.001) and high level of glucose (OR 1.08, 95%CI 1.01-1.16, *p=*0.021) were independent risk factors for ICAS in the present study. These associations were still robust after controlling for covariates.

### ApoB/AI ratio and extent of steno-occlusion lesions

Table 3 manifests that increased apoB/AI ratio is associated with the extent of steno-occlusion lesions. (Supplementary Figure 2B shows the distribution of subjects according to quartiles in three groups). Comparing the no-ICAS group and the group of 1-3 stenosis arteries, the discrepancy was significant (*p* <0.001), suggesting that the high level of apoB/AI ratio is a valuable risk factor for atherosclerotic stenosis. However, the comparison between the group of 1-3 stenosis arteries and the group of 4 or more stenosis arteries (*p* =0.081) failed to demonstrate the association.

**Table 3:** Comparisons of apoB/AI ratio in different groups according to steno-occlusion lesions.

	**No ICAS**	**ICAS groups**		
ICAS numbers	0 (n=1614)	1–3 (n=537)	4 or more (n=59)	P^*^
ApoB/AI Ratio, mg/dl	0.78±0.26	0.85±0.27	0.91±0.25	<0.001

ICAS, intracranial atherosclerotic stenosis;

p^*^, significantly different in both comparisons of no-ICAS group with the group of 1-3 stenosis arteries or the group of 4 or more stenosis arteries.

## DISCUSSION

The present study expanded previous researches via observing the association of apoB/AI and ICAS in stroke patients and meanwhile comparing the same indicators in non-stroke controls for contrast. The results showed that individuals with ICAS had higher apoB and lower apoAI compared to those without ICAS in both stroke and non-stroke groups. Several studies have mentioned that apoAI as a marker of antioxidant properties played a critical role in anti-thrombus that could protect intracranial artery, and apoAI deficiency might be the pathomechanism accountable for infarct injury from intracranial vascular bed [10, 14, 16, 17]. ApoB was generally regarded as a marker of oxidative and atherogenic properties and the degradation of plasmatic LDL-c could prevent advanced atherosclerotic lesion progression [18]. The ratio of apoB/AI best reflects the status of cholesterol transport to and from peripheral tissues [19]. Overdose lipid would deposit under the endangium of arteries [17, 20]. With the apoB/AI ratio increases, more cholesterol is likely to be deposited in the arterial wall, thereby accelerating atherogenesis and increasing vascular risk [14, 21]. Our results also showed that in the stroke group patients with higher apoB/AI ratio were at a 2.80-fold greater risk of suffering atherosclerotic stenosis compared to those with lower apoB/AI ratio, which supported the view that the high level of apoB/AI ratio was a valuable risk factor for ICAS among stroke patients. Increase apoB/AI ratio has been demonstrated as an independent risk factor for ischemic stroke. ICAS is an important risk factor for ischemic stroke, and its progression is strongly associated with an increased risk of ischemic events in future. As the apoB/AI ratio increased, the risk of suffering stenosis increased, which may raise the morbidity of ischemic stroke. Whether there is a cooperative action between apoB/AI ratio and ICAS on ischemic stroke needs further investigation. The apoB/AI ratio has the same value in non-stroke controls. Compared to individuals with lower apoB/AI ratio, the risk of suffering ICAS was 3.38-fold greater in those with higher apoB/AI ratio. Meanwhile, the highest quartile and the third quartile had a 2.00-fold risk and a 1.71-fold risk respectively, compared with the lowest quartile.

The association between apoB/AI ratio and extent of steno-occlusion lesions provided more evidences. The differences were significant in the apoB/AI ratio in both comparisons of no-ICAS group with the group of 1-3 stenosis arteries or the group of 4 or more stenosis arteries. That also proved that regardless of whether participants had or did not have ischemic stroke, apoB/AI ratio was still an independent risk factor for ICAS. But the comparison between the other two groups failed to demonstrate that apoB/AI ratio was a valuable risk factor for the extent of steno-occlusion lesions. The current research also failed to demonstrate that a high LDL-c level was an independent predictor of ischemic stroke or ICAS, which was consistent with several previous studies [20, 22] suggesting that high apoB/AI ratio had a closer association with increased artery intima-media thickness than any other lipids or lipid ratios. Hence, it appears to be a considerable advantage to apply apolipoproteins into clinical practices [23].

The study also has some limitations. Firstly, the present research was not conducted with a purpose of accessing all the risk factors associated with cerebral atherosclerotic stenosis. It was designed to testify whether apoB/AI ratio was an independent risk factor for stenosis irrespective of other risk factors such as visceral obesity and so on. The potential clinical roles of these apolipoproteins in atherosclerosis or ischemic stroke warrant further study. Secondly, long term follow-up for the morbidity of ischemic stroke in non-stroke group was required to further prove that high level of apoB/AI ratio might increase the risk of ischemic stroke through accelerating ICAS.

## CONCLUSIONS

The current study demonstrated that the apoB/AI ratio was an independent risk factor for ICAS in both stroke and non-stroke Chinese individuals, suggesting the effects of apoB/AI ratio on increasing the risk of ICAS were the same between stroke and non-stroke individuals. We found that the risk of suffering ICAS will increase when apoB/AI ratio increased.

## MATERIALS AND METHODS

### Study population

Study subjects were prospectively recruited from January 2014 to June 2018 among patients referred to department of neurology of Qingdao Municipal Hospital for suspected stroke and individuals underwent comprehensive health screening, including brain MRI and magnetic resonance angiography (MRA), at health screening center of Qingdao Municipal Hospital. The non-stroke controls are participants from health screening center who did not have serious health problems as well as TIA or acute ischemic stroke confirmed by neuroimaging. We excluded individuals who: (1) less than 40 years old; (2) underwent incomplete vascular imaging and laboratory tests; (3) had been on statin or fibrate before admission because these drugs could affect the apoB/AI level; (4) had atrial fibrillation, cardiac embolism, vascular disease and had underwent replacement; (5) had intracranial and external artery dissection, arteritis, moyamoya disease, muscular fiber dysplasia; (6) had infection, nausea, tumor, chronic liver disease and renal insufficiency; (7) had history of ischemic stroke. Written informed consent form was obtained from all participants or their legal representatives. This study was approved by the Institutional Ethics Committees of Qingdao Municipal Hospital.

Finally, 1072 non-stroke controls and 1138 stroke patients were included in this study for the analysis (Flowchart of the screening process about the included participants was listed in the Supplementary Figure 1).

### Assessment of clinical risk factors

Participants were assessed according to stroke registry information, including demographic profiles, living habits and biomarker data of stroke or vascular risk [24]. Age, sex, weight, body mass index (BMI), history of hypertension, history of diabetes, alcohol consumption, smoking, and high level of glucose were recorded. Before the measurement of blood pressure, subjects were required to rest for at least five min. All subjects were measured at least twice to obtain stable blood pressures. Systolic blood pressure over 140 or diastolic blood pressure over 90 mmHg were regarded as hypertension [25]. Diabetes mellitus was defined as a level of fasting plasma glucose≥7.0mmol/L. Alcohol abuse was defined as moderate to severe alcohol consumption in social history (>168g/week) [24]. We also considered those who smoked regularly at least one cigarette per day at the time of presentation as current smokers [24].

### Laboratory measurements

Blood samples of all the participants were drawn after an overnight fast during 24 hours of stroke onset. Serum levels of HDL-c and LDL-c were assayed by enzymatic techniques (Beckman Coulter (AU5800), USA), and the apoB and apoAI levels were assayed by immunonephelometry using a BN II analyzer (Siemens Healthcare, Marburg, Germany) [16]. Some studies have demonstrated that apoB/AI ratio could keep stable within a time interval from stroke onset to the following four weeks [16, 26]. Therefore, the time interval from stroke onset to the measurements of apoB and apoAI affects the validity of the findings scarcely.

### Assessment of ICAS

All enrolled participants were evaluated through neuroimaging and vascular imaging, including brain MRI (magnetic resonance imaging) [16] and 3D time-of-flight MRA (magnetic resonance angiography). Brain MRI and MRA were performed with a 3.0 tesla unit. Diffusion-weighted MRI (DWI) was used to define the presence of acute ischemic stroke, and MRA was performed to determine the location and extent of ICAS. Doppler ultrasonography and contrast-enhanced MRA were used to evaluate the extracranial carotid arteries excluding a cardiac or carotid artery source of embolus that might be another etiology of stroke. [2].

The intracranial arteries we evaluated included the intracranial segment of internal carotid and vertebral arteries, basilar artery, the proximal segment of middle cerebral artery (M1, M2), anterior cerebral artery (A1, A2), and posterior cerebral artery (P1, P2). We defined ICAS as atherosclerotic stenosis >50% or the occlusion of the above main intracranial arteries [24, 27]. For the accuracy to prove the association between apoB/AI ratio and ICAS, we exclude the extracranial segment vascular stenosis (Supplementary Figure 3 and Figure 4, showed several representative MRI and ultrasound pictures of ICAS). Two experienced radiologists who were blinded to clinical data interpreted the angiographic features, and disagreements over the presence of stenosis on angiography were solved by consensus.

### Subject grouping

All participants were categorized into four subgroups based on an angiographic study, including (1) stroke with artery stenosis (the SICAS group, ischemic stroke with significant atherosclerotic stenosis in the intracranial arteries); (2)stroke without artery stenosis (the SNCAS group, ischemic stroke without significant atherosclerotic stenosis); (3)non-stroke with artery stenosis (the NICAS group, no ischemic stroke but with significant atherosclerotic stenosis); (4)non-stroke without artery stenosis (the NNCAS group, no ischemic stroke without significant atherosclerotic stenosis). To assess the association between apoB/AI ratio and extent of intracranial atherosclerotic steno-occlusion lesions, all participants were further divided into three groups according to number of affected intracranial arteries: no lesion of ICAS (n=1612), 1-3 intracranial stenosis (n=537), 4 or more intracranial stenosis (n=59).

### Statistical analysis

All parameters were presented as mean±SD (standard deviation) or proportions appropriately. Wilcoxon test was used to compare intergroup difference for continuous variables, while Chi-squared test was used to examine categorical variables.

We compared baseline variables between stroke and non-stroke groups, SICAS and SNCAS groups, NICAS and NNCAS groups by the above methods. We also evaluated the association between apoB/AI ratio and steno-occlusion lesions among groups using Kruskal- Wallis test. Multivariate logistic regression analyses were performed to explore whether level or quartile of apoB/AI ratio was independently associated with the presence of ICAS, all covariates were included in the model. The lowest quartile was defined as the reference group. Multivariate logistic regression analysis was further performed to measure the relationship between apoB/AI ratio and ischemic stroke. Results are presented as ORs with their 95% CIs. The tests of interactions between HDL-c, LDL-c and apoB/AI ratio were conducted in both stroke and non-stroke groups. We also conducted the power analysis using G*Power 3.1.9.4. The group sample sizes of 359 and 779 in stroke patients achieve 85% power and the group sample sizes of 239 and 833 in non-stroke controls achieve 99% power to detect the difference of apoB/AI between ICAS and NCAS groups. Overall, other statistical tests were conducted using R software version 3.4.2, and the significance level was set at *p* <0.05 for all data.

## Supplementary Material

Supplementary Figures
