# Supplementary material for: Apolipoprotein B/AI ratio as an independent risk factor for intracranial atherosclerotic stenosis
Source: Aging (Albany NY). 2019 Sep 3;11(17):6851–62. doi: 10.18632/aging.102216 (PMC6756877; doi:10.18632/aging.102216)
Supplement: Supplementary Figures [file aging-11-102216-s001.pdf]

SUPPLEMENTARY FIGURES

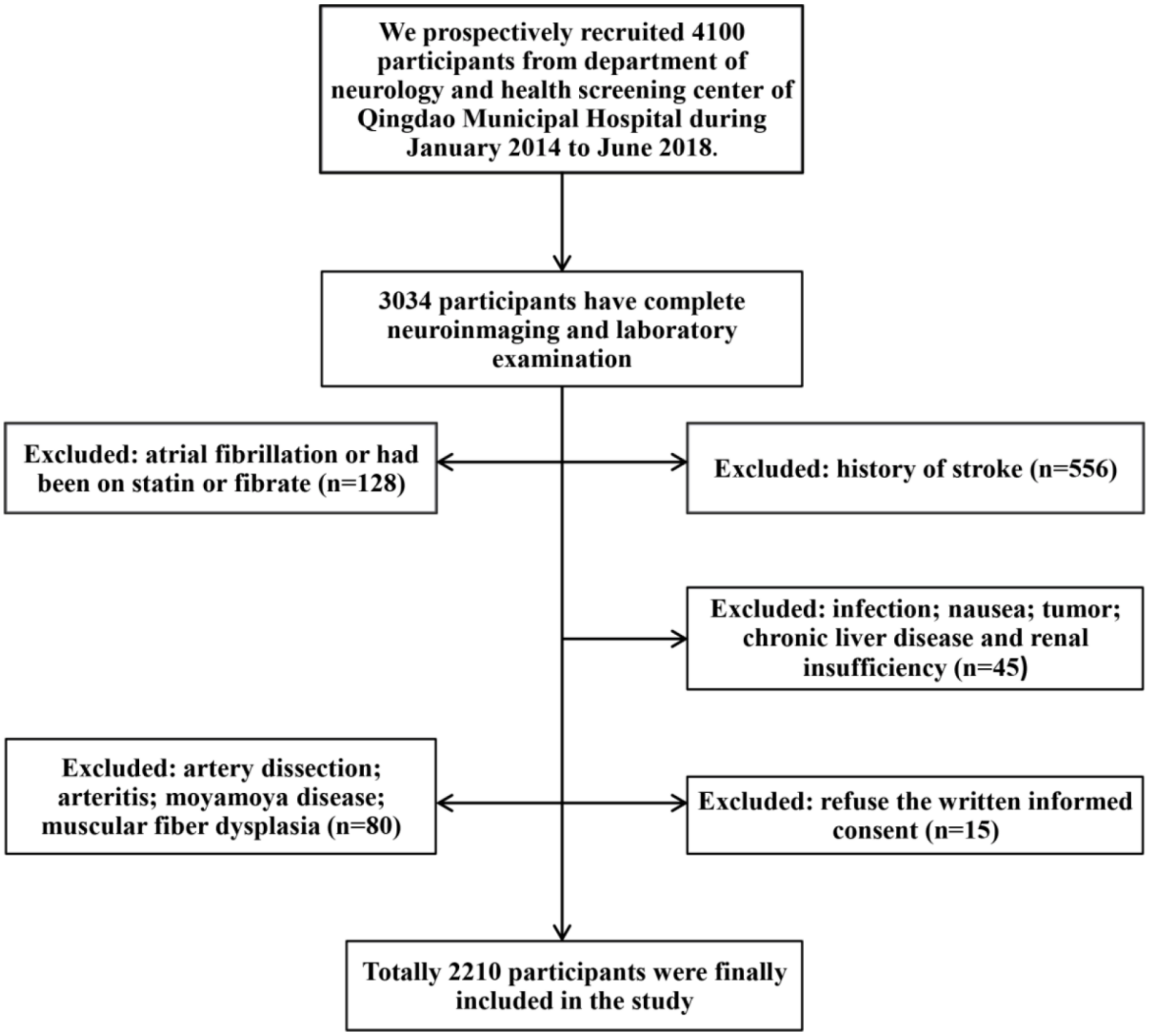

Supplementary Figure 1. The flowchart of the screening process about the included participants.

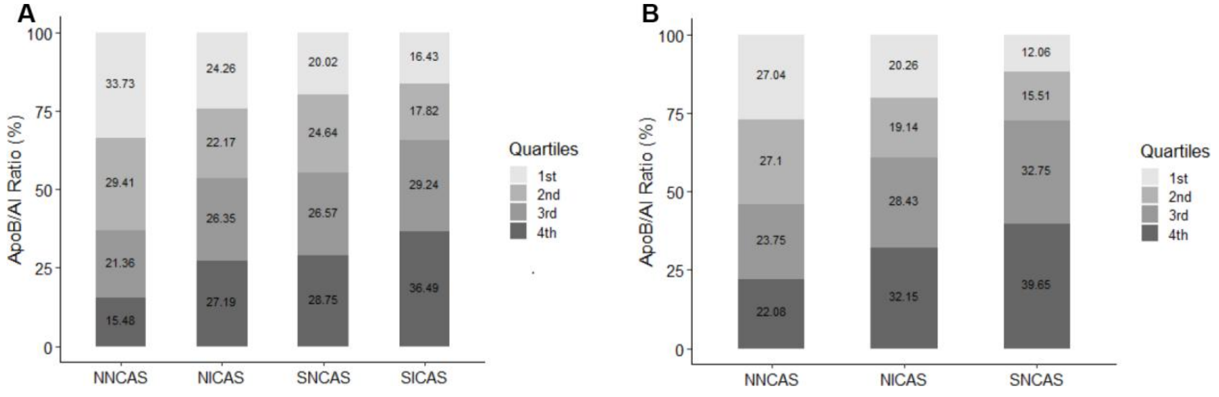

Supplementary Figure 2. Distributions of 4 study groups (**A**) extent of ICAS lesions (**B**) according to the apoB/AI ratio quartiles. Values represent percentages of patients. As the apoB/AI ratio quartiles increased, the presence of ischemic stroke, ICAS and extent of steno-occlusion lesions increased. ICAS, intracranial atherosclerotic stenosis.

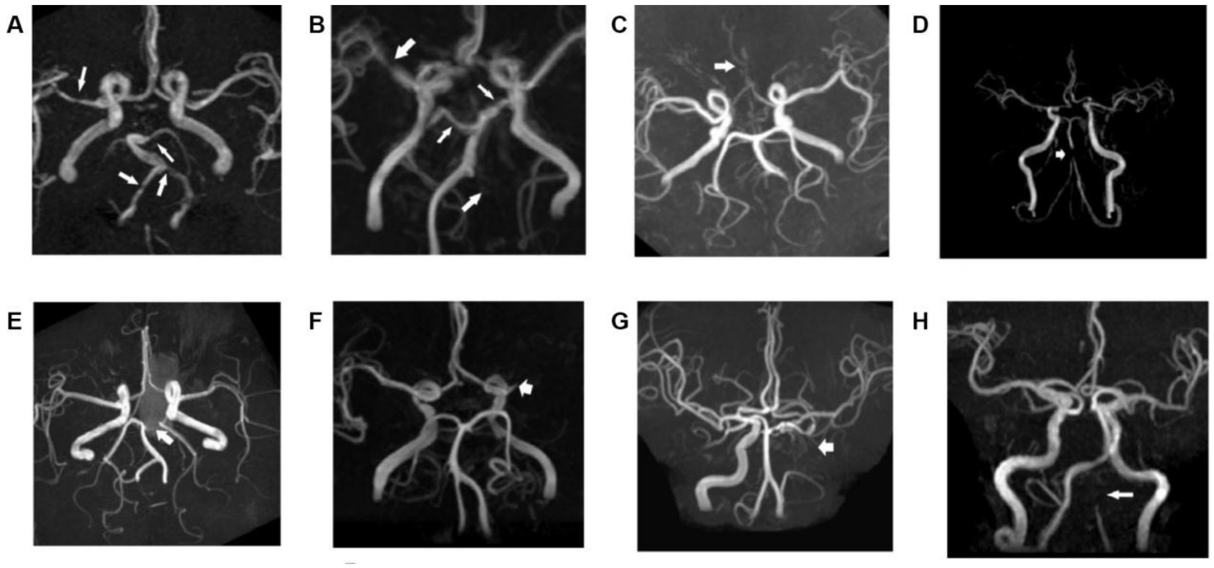

Supplementary Figure 3. Several pictures for the MRI. (**A**) MRA showing stenotic lesions at right middle cerebral artery (61.7%), left posterior cerebral artery (55%) and right and left vertebral arteries (65% and 50%). (**B**) MRA showing stenotic lesions at right middle cerebral artery (70%), left vertebral artery (occlusion) and right and left posterior cerebral artery (55% and 55%). (**C**) MRA showing stenotic lesions at right and left anterior cerebral arteries (occlusion). (**D**) MRA showing stenotic lesion at basilar artery (almost occlusion). (**E**) MRA showing stenotic lesion at left posterior cerebral artery (90%). (**F**) MRA showing stenotic lesion at left middle cerebral artery (occlusion). (**G**) MRA showing stenotic lesion at internal carotid artery (occlusion). (**H**) MRA showing stenotic lesion at left vertebral artery (almost occlusion).

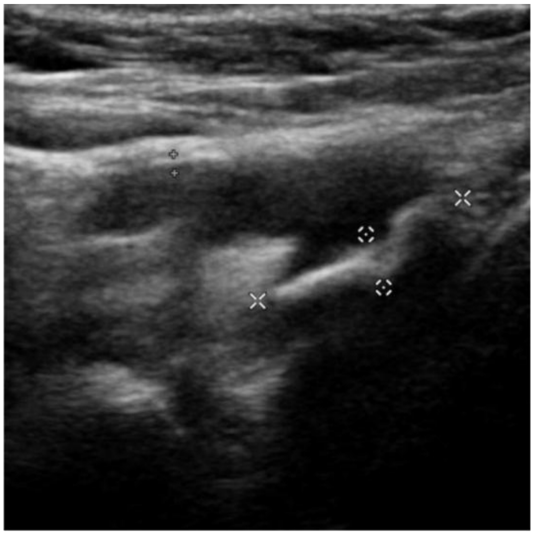

Supplementary Figure 4. The Doppler sonography showed the formation of atheromatous plaques in internal carotid artery.
